# Improving the delivery and efficiency of fungus-impregnated cloths for control of adult *Aedes aegypti* using a synthetic attractive lure

**DOI:** 10.1186/s13071-018-2871-z

**Published:** 2018-05-04

**Authors:** Adriano R. Paula, Leila E. I. Silva, Anderson Ribeiro, Tariq M. Butt, Carlos P. Silva, Richard I. Samuels

**Affiliations:** 1 Department of Entomology and Plant Pathology, Universidade Estadual do Norte Fluminense Darcy Ribeiro, Campos dos Goytacazes, RJ 28013-602 Brazil; 2 Department of Biosciences, College of Science, Swansea University, Singleton Park, Swansea, SA2 8PP UK; 3 Departamento de Bioquímica, Universidade Federal de Santa Catarina, Florianópolis, 88040-900 Brazil

**Keywords:** Fungal virulence, Intra-domicile conditions, Attractive lure, Traps, Vector, Insect, Dengue, Zika

## Abstract

**Background:**

Entomopathogenic fungi are highly promising agents for controlling *Aedes aegypti* mosquitoes. Deploying fungus-impregnated black cloths in PET traps efficiently reduced *Ae. aegypti* female survival rates under intra-domicile conditions. With the aim of further increasing the effectiveness of the traps, the addition of attractive lures to fungus-impregnated traps was evaluated.

**Methods:**

Black cloths were suspended inside 2 l plastic bottles called “PET traps”. These traps were placed in rooms simulating human residences. The first experiments evaluated the attraction of mosquitoes to PET traps with black cloths covered in adhesive film with and without synthetic lures (Atr*Aedes*™). Traps were left in the test rooms for either 24 or 48 h. The attractiveness of the lures over time was also evaluated. The efficiency of PET traps with fungus-impregnated black cloths associated with lures was compared to that of traps without lures.

**Results:**

The highest percentage of captured mosquitoes (31 and 66%) were observed in PET traps with black cloths covered in adhesive film + attractive lure maintained in test rooms for 24 h and 48 h, respectively. Black cloths covered in adhesive film captured 17 or 36% of the mosquitoes at 24 h and 48 h, respectively. The attractiveness of the lures fell gradually over time, capturing 37% after 5 days on the bench and 22% of the mosquitoes after 30 days exposure to ambient conditions. Associating attractive synthetic lures with black cloths impregnated with *M. anisopliae* placed in test rooms for 120 h reduced mean survival to 32%, whilst black cloths impregnated with *M. anisopliae* without lures resulted in a 48% survival rate. Using *Beauveria bassiana* in the traps resulted in a 52% reduction in mosquito survival, whilst combining *Beauveria* and Atr*Aedes* resulted in a 36% survival rate. PET traps impregnated with fungus + Atr*Aedes* resulted in similar reductions in survival when left in the rooms for 24, 48, 72 or 120 h.

**Conclusions:**

Atr*Aedes* increased attractiveness of PET traps with black cloths under intra-domicile conditions and when associated with *M. anisopliae* or *B. bassiana*, significantly reduced *Aedes* survival. This strategy will reduce the number of PET traps necessary per household.

**Electronic supplementary material:**

The online version of this article (10.1186/s13071-018-2871-z) contains supplementary material, which is available to authorized users.

## Background

The vector control methods currently deployed in Brazil are failing to avert epidemics of mosquito-borne diseases such as dengue, Zika, chikungunya and the most recent outbreak of yellow fever. The mainstay of many governmental mosquito control programs is the application of synthetic insecticides using peridomestic space spraying *via* ultra-low-volume technology. However, there is no evidence to support the effectiveness of this technique as a stand-alone intervention [1]. Peridomestic space spraying should only be employed as part of an integrated vector management strategy.

New innovative approaches are urgently needed to implement integrated vector control strategies, including the use of insecticides during epidemics, with the aim of maintaining mosquito populations at levels which significantly reduce the risk of rapid transmission of diseases. Alternative strategies such as the use of transgenic mosquitoes, sterile male release and biological control measures could provide important contributions to combat insect vectors.

The Oxitec Ltd. program involving the mass release of transgenic “sterile” *Aedes aegypti* males (http://oxitec.com/friendly-mosquitoes/) aims to eliminate *Ae. aegypti* mosquitoes; however, the long term effects on the ecosystem will be difficult to predict and the influx of mosquitoes from neighboring populations could nullify the benefits of this program. Another alternative approach called “Our Challenge, the World Mosquito Programme” (http://www.eliminatedengue.com/program) is currently being tested in Brazil, which involves the release of females infected with *Wolbachia* bacteria into the environment. *Wolbachia-*infected females are refractory for the dengue virus [2], therefore this program aims to reduce dengue transmission without reducing the mosquito population. The establishment of *Wolbachia-*infected mosquito populations depends on the dominance of these populations over wild type populations and that non-infected mosquitoes entering areas with *Wolbachia-*infected mosquitoes, do not repopulate that area. The release of male mosquitoes carrying a dominant lethal gene or the release of *Wolbachia-*infected mosquitoes results in females ovipositing non-viable eggs and, by alleviating intense larval competition, could result in an overall increase in wild type larval survival to the adult stage [3].

The deployment of biological control agents such as entomopathogenic fungi is an important alternative or complement to conventional control methods. Many studies have shown the potential of entomopathogenic fungi for the control of insect vectors [4–6]. One of the main advantages of using entomopathogenic fungi against insect pests and vectors is that these fungi can infect all stages of the insects’ life-cycle, from egg to adult [7–9]. Moreover, no cases of resistance of insects to entomopathogenic fungal infections have been reported to date.

Entomopathogenic fungi have the added advantage of being spread by autodissemination *via* contact of an infected mosquito with other mosquitoes, for example, during mating. It has been shown that male *Ae. aegypti* infected with either *Beauveria bassiana* or *Metarhizium anisopliae* can transmit the fungus to females during mating [10, 11].

A bio-rational approach for mosquito control could be the use of biological agents associated with lures which attract adult mosquitoes to “traps” where they become infected (lure and kill), rather than applying insecticides or biological control products against mosquito larvae at putative breeding sites.

A range of sophisticated mosquito traps is now commercially available for population monitoring or as control measures, although their cost and logistics could be prohibitive for implementation on a large scale. The BG Sentinel trap (Biogents, Regensburg, Germany) is a very useful monitoring tool but may not be viable for controlling mosquito populations in the field. The current cost of purchasing one of these traps is around €200 to €300. The trap also requires a power source, supplied by either a motorcycle battery or 110 V household socket. A recent study in Manaus in the north of Brazil showed that mass trapping using the BG Sentinel significantly reduced *Aedes* populations; however, the incidence of dengue fever was not significantly altered [12].

A trap combining an entomopathogenic fungus and insecticide is being marketed in over 25 countries by “In2Care” , Wageningen, the Netherlands (http://www.in2care.org). The trap attracts female mosquitoes (using a yeast tablet attractant) and contains a landing surface impregnated with *B. bassina* and a synthetic larvicide (pyriproxyfen) added to the water in the trap. Females lay their eggs inside the trap and become contaminated with both the fungus and insecticide. Larvae hatching in the trap are killed by the insecticide (100% larval mortality was observed) and females leaving the trap carry the insecticide to other oviposition sites and subsequently kill larvae (> 90%) present at those sites. Females become infected and are killed by the fungus [13].

A solar-powered trap, also containing a combination of control agents, has been tested in Africa for the control of *Anopheles arabiensis* [14]. These authors obtained high contamination rates of malaria mosquitoes even when humans were competing with the attractiveness of the traps. This demonstrated the potential of these traps for controlling outdoor-biting malaria vectors, either by reducing their survival or directly killing host-seeking mosquitoes. Furthermore, the traps can be used in combination with larvicides such as pyriproxyfen, which mosquitoes carry to aquatic habitats, thus controlling larval populations as mentioned above.

Simple lure and kill strategies such as impregnating clay water storage pots with fungal spores [15], or applying fungi to house screens, such as eave curtains, have been shown to be promising delivery tools for infecting mosquitoes that come into contact with the conidia during host seeking or oviposition behavior [16].

We have developed a simple and cheap lure and kill adult trap based on the use of a fungus-impregnated black cloth suspended in a PET bottle. We have shown the attractiveness of these traps to female *Ae. aegypti* and the efficiency of these traps in reducing adult survival under intra-domicile conditions [17]. In the current study, the use of a synthetic lure in combination with fungus-impregnated black cloths was shown to improve the efficiency of the traps in reducing adult female survival. This PET trap is a low-cost user-friendly system which we are now testing in human residences.

## Methods

### Maintenance of insect colonies

*Aedes aegypti* larvae were obtained from field collected mosquito eggs. Eggs were collected using “ovitraps” placed around the University campus. Only F1 adults were used in all experiments. Larvae were maintained in plastic trays (approximately 80–100 larvae per 100 ml) and fed on freshly ground and autoclaved commercial mouse food (Nuvilab, São Paulo, Brazil; 0.05 g per l) until reaching the pupal stage. Pupae were separated into water-filled beakers and transferred to cages before adult emergence. Adults were maintained in cages with 10% sucrose wick feeders. Recently emerged (2–3 days old) females that had been maintained in cages with males, were separated for use in all experiments.

### Attractiveness of PET trap with and without lures under intra-domicile conditions

Four treatments were used to evaluate the attractiveness of PET traps with and without Atr*Aedes* lures (Agrisense Ltd, Cardiff, UK). The PET traps were constructed from a 2 l clear transparent PET bottle (Additional file 1: Figure S1a), with a lateral cut (17 × 8 cm) to facilitate mosquito access. Capture rates of traps were evaluated using adhesive transparent film (16 × 7 cm; Agrisense Ltd), suspended in the traps using a steel wire. The second treatment was carried out by attaching an Atr*Aedes* lure to the bottom of the adhesive film (Additional file 1: Figure S1b: PET trap with lure). The third treatment was performed to test the attraction of PET traps using black cotton cloths (16 × 7 cm) covered with adhesive film. The fourth treatment tested the combination of PET traps with black cotton cloths covered in adhesive film and an Atr*Aedes* lure attached to the bottom of the cloth (Additional file 1: Figure S1c: PET trap with lure and black cloth). In order to increase the trap stability, the bottom of the PET trap was filled with plaster of Paris. The number of mosquitoes captured on the adhesive films over a 24 and 48 h period were evaluated.

Experiments were carried out in rooms simulating human residences. Four identical rooms built on the outside of the insectary of the State University of North Fluminense [each room: 4 m (L) × 3 m (W) × 2.2 m (H)] with ceramic floor tiles, plastered walls painted white, PVC lined ceiling and a wooden door (200 × 80 cm) with an built-in mosquito screen (150 × 40 cm) were used for these experiments (Additional file 2: Figures S2a, b, c). These rooms were similar to many bedrooms found in this region of Brazil. A constantly circulating current of air in the room was created by an extractor fan in the ceiling of each room. Items of furniture were placed in each room (2 chairs and 1 desk). The rooms were prepared for experiments by wiping down all surfaces with disinfectant (10% bleach) and then tap water. Two wick feeders were placed in each room with 30 ml of 10% sucrose and were not refilled during the period of the experiments. Each wick feeder was placed in the middle of Petri dishes with talcum powder (Vetec, São Paulo, Brazil) to avoid attracting domestic ants. Dataloggers (ESCORT RH iLog, Buchanan, USA) were used to monitor temperature and humidity during all experiments in the rooms (temperature, max: 27.9 °C, min: 24.5 °C; relative humidity RH, max: 75.2%, min: 62.8%). Fifty female mosquitoes were released into each room with one PET trap per room and the number of mosquitoes captured by the adhesive films was counted at 24 and 48 h. The 24 h count was carried out by rapidly entering the rooms and observing the number of mosquitoes stuck to the adhesive films. At 48h, any mosquitoes that had not been trapped by the adhesive films were killed using a hand held sprayer with 70% alcohol. The four treatments were carried out simultaneously and repeated three times.

### Attractiveness of Atr*Aedes* lures over time

These experiments were carried out to verify the attractiveness of the Atr*Aedes* lures when left for different time periods under normal ambient conditions before being placed in PET traps with adhesive films. The lures were left in open Petri dishes for a total of thirty days. The previous experiments demonstrated capture rates when using lures which had been stored at 4 °C (time zero), therefore here lures were tested following 5, 10, 15, 20, 25 and 30 days when maintained under ambient conditions (approximately 25 °C and 70% RH). Following each time period, the lures were attached to PET traps as described in the previous section and one trap was placed in each test room. Fifty female mosquitoes were released into each room and the capture rates were observed at 24 h and 48 h (as stated previously).

### Mosquito survival rates in the presence of PET trap with fungus-impregnated cloths in combination with Atr*Aedes* lures under intra-domicile conditions

#### Fungal isolate and preparation of suspensions

The isolate of *M. anisopliae* used here was obtained from the collection at the Escola Superior de Agricultura “Luiz de Queiroz” (ESALQ 818) in Piracicaba (São Paulo), which had been previously demonstrated to have high virulence against adult *Ae. aegypti* [9]. The isolate of *B. bassiana* used here, CG24, was obtained from the EMBRAPA-CENARGEN collection in Brasilia.

Fungi were cultured on Sabouraud dextrose agar (dextrose 10 g, peptone 2.5 g, yeast extract 2.5 g, agar 20 g in 1 l H_2_0) at 27 °C for 15 days. Conidia were then harvested directly from the solid media using a spatula and suspended in Tween 80 (TW) (0.05 % in sterile distilled water). This suspension was then inoculated into 250 ml conical flasks containing 25 g of par-boiled rice + 10 ml distilled water, which had been previously sterilized (121 °C for 15 min). Following inoculation, the flasks were incubated at 27 °C for up to 15 days. Following this time, the conidia were transferred to paper bags and humidity reduced using a forced-air incubator at 35 °C for 3 days before separating the conidia from the rice grains using an MR-5 Mycoharvester® (ACIS, Tavistock, UK). Conidia were collected and the dry-weight to conidial concentration ratio was calculated by suspending known weights of dry conidia in TW and the concentration determined using a Neubauer chamber. The fungal concentration used in all tests was 1 × 10^8^ conidia ml^-1^. Viability tests were carried out by plate counting and only batches with > 90% germination were used in experiments.

Black cloths (16 × 7 cm) were autoclaved for 15 min at 1 atm (121 °C) and immersed in 200 ml of suspensions of fungi formulated in 0.05% TW For controls, the black cloths were impregnated with TW only. The black cloths impregnated with fungal suspensions were hung on a clothes-line to dry for 16 h in a room with an average temperature of 26 °C, 71% RH. The black clothes were then suspended in PET traps.

Four treatments were carried out simultaneously in test rooms simulating human residences (as described above). The first test was carried out deploying one PET trap with a fungus-impregnated cloth associated with an attractive lure. The second treatment investigated the effect on mosquito survival of a PET trap with a fungus-impregnated cloth only. The control treatments investigated any possible effects on mosquito survival due to the presence of PET traps with black cloths only and black cloths with Atr*Aedes* lures.

Fifty female mosquitoes were released into each room and after five days (120 h) a BG-Sentinel™ trap with a BG-Lure attractant (Biogents Ltd.) was quickly placed in each room and 24 h later the number of captured mosquitoes was recorded. The rooms were then inspected for any mosquitoes that had failed to be captured. Each experiment was carried out three times.

#### Effect on mosquito survival when traps were maintained in rooms for different time periods

One PET trap was placed in each room and then fifty female mosquitoes were released into the rooms. After 24, 48 and 72 h, the traps were carefully removed from the room without allowing any mosquitoes to escape. After a total of 5 days, live mosquitoes were captured using a BG-Sentinel™ trap. For the 120 h exposure time, the PET trap was not removed but a BG-Sentinel™ trap was placed in the room as stated above to capture live mosquitoes. In these experiments black cloths were impregnated with *M. anisopliae*. Four treatments were used: (i) PET traps + fungus + lure; (ii) PET traps + fungus; (iii) PET traps with black cloths + lure; and (iv) PET traps with black cloths only.

### Statistical analysis

All experiments were carried out three times with 50 insects per treatment or per control group. A one-way analysis of variance (ANOVA) was used to compare the capture on adhesive films or survival of mosquitoes following the different treatments. Survival rates were calculated as the percentage of mosquitoes captured in the BG-Sentinel™ traps at the end of the experiments, when compared to number initially released into each room (50 females). When a significant effect of groups was recorded, data were further analyzed by Duncan’s *post-hoc* test with *P* < 0.05 as the criterion for statistical significance.

## Results

### Preliminary evaluation of mosquito attraction to PET traps

These experiments were carried out to verify the attractiveness of PET traps using adhesive films to capture mosquitoes (Table 1). When traps were tested with adhesive film only, very low numbers of mosquitoes were captured over 24 and 48 h (3 and 6%, respectively). The next experiment evaluated the capture rates when placing an Atr*Aedes* lure on the adhesive film. An increase in the number of mosquitoes stuck to the film was recorded, with 10% capture at 24 h and 22% at 48 h. Although there was no statistically significant difference between capture rates of films with and without lures at 24 h, a significant difference was seen when comparing these two treatments at 48 h (*F*_(1, 5)_ = 1515, *P* < 0.01). The attractiveness of black cloths covered in adhesive film was then evaluated. These traps captured 17% of the mosquitos at 24 h and 36% at 48 h. The capture rates were significantly higher than films + Atr*Aedes* at 48 h (*F*_(1, 5)_ = 5.71, *P* < 0.01), showing the importance of the black cloths in attracting mosquitoes. However, the highest percentage of captured mosquitoes (31%) was observed in PET traps with black cloths covered in adhesive film + attractive lure maintained in test rooms for 24 h, when compared to all other treatments. At 48 h, the highest number of captured insects (66%) was observed when combining adhesive film + black cloths + attractive lure.

**Table 1:** Percentage of mosquitoes captured on adhesive films in PET traps with black cloths or black cloths covered in adhesive films plus attractive lures over a 24 or 48 h time period

Treatment	Mosquitoes captured over 24 h (mean % ± SD)	Mosquitos captured over 48 h (mean % ± SD)
Adhesive film only	3.3 ± 1.5 c	6.7 ± 1.5 d
Adhesive film + attractant	10.6 ± 2.5 bc	22.6 ± 3.2 c
Black cloth + adhesive film	17.3 ± 1.6 b	36.0 ± 3.6 b
Black cloth + adhesive film + attractant	31.3 ± 3.1 a	66.6 ± 1.5 a

Results followed by different letters indicate significant differences between treatments during one time period when using Duncan’s *post-hoc* test (5% probability)

### Attractiveness of Atr*Aedes* over time

**Fig. 1:**
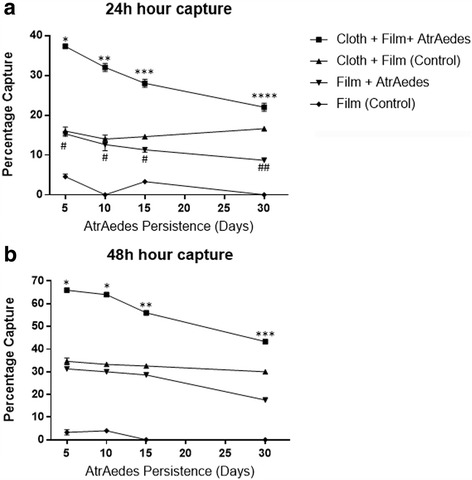
**a** Attractiveness of Atr*Aedes* lures as measured by capture rates of *Aedes aegypti* when released in a room with PET traps + adhesive films over a 24 h period. **b** Attractiveness of Atr*Aedes* lures over a 48 h period. Note for both figures: different numbers of symbols indicate significant differences (5% level) between the capture rates for one type of PET trap when comparing attractiveness of lures over time

These tests were carried out to evaluate the attractiveness of the lures over time, as this information is not available in the literature. The capture rates for “time zero” were evaluated in the previous experiment when lures were maintained at 4 °C before use. Lures that had been left on the bench under normal ambient conditions for 5 days attracted 37% of the mosquitoes over a 24 h period when associated with black cloths covered in adhesive film (Fig. 1a). The attractiveness of the lure attached to black cloths was reduced to a 22% capture rate when the lures had been left on the bench for a 30-day period. There was a significant reduction in capture rates during this experiment when comparing each time point (*F*_(4, 14)_ = 48.09, *P* < 0.01). However, this was not the case when comparing capture rates of PET traps with adhesive film + Atr*Aedes* only. Although these traps captured lower numbers of mosquitoes, a significant reduction in capture rates was only seen when the lures were deployed after a 30 day period on the bench (*F*_(4, 14)_ = 11.72, *P* < 0.01) (Fig. 1a). A similar pattern was observed for experiments evaluating capture rates at 48 h (Fig. 1b).

### Efficiency of PET traps impregnated with entomopathogenic fungi associated with lures

In these experiments the survival of mosquitoes released in the test rooms was evaluated after a 5-day period (Table 2). Associating attractive synthetic lures with black cloths impregnated with *M. anisopliae* in test rooms resulted in 32% survival, whilst black cloths impregnated with *M. anisopliae*, but without attractive lures, resulted in a 48% survival rate over the 5-day evaluation period. The addition of lures to the PET trap resulted in a statistically significant reduction in survival (*F*_(3, 11)_ = 64.87, *P* < 0.01). Control survival rates were approximately 80%.

**Table 2:** Survival of mosquitoes exposed to PET traps with fungus impregnated black cloths when associated with attractive lures in test rooms over a 5-day period

Treatment	Survival (mean % ± SD)	
	*M. anisopliae*	*B. bassiana*
Black cloth + fungus + lure	32.6 ± 1.2 a	36.0 ± 2.1 a
Black cloth + fungus	48.0 ± 3.6 b	52.0 ± 4.0 b
Black cloth + lure	80.0 ± 2.1 c	83.0 ± 1.5 c
Black cloth only	82.0 ± 2.1 c	78.0 ± 2.3 c

Results followed by different letters indicate significant differences between treatments when using Duncan’s *post-hoc* test (5% probability)

Similar results were seen when using black cloths impregnated with *B. bassiana* (Table 2). The lowest survival rates were seen when combining *B. bassiana*-impregnated cloths with the Atr*Aedes* lure, with 36% of the mosquitoes surviving after 5 days. PET traps with the fungus alone caused a 52% reduction in survival. The differences between survival rates for all the treatments here were significant (*F*_(3, 11)_ = 52.4, *P* < 0.01).

PET traps were then left in the rooms for different time periods with the aim of investigating if exposure times of less than 5 days would still result in a significant reduction in survival (Table 3). Interestingly, PET traps impregnated with fungus associated with the lures caused similar reductions in survival when left in the rooms for 24, 48, 72 or 120 h (*P* > 0.01), whereas PET traps impregnated with fungus but without lures, were ineffective at 24 h, resulting in a survival rate of 76% which was not significantly different from the controls (*P* > 0.01). However, increasing the time the fungus-impregnated traps were left in the room resulted in a steady and significant reduction in survival rates when comparing exposure times to the fungus (*F*_(3, 11)_ = 20, *P* < 0.01), although there was no significant difference between 72 h (56% survival) and 120 h (47% survival).

**Table 3:** *Aedes aegypti* survival rates (%) following different exposure times to PET traps impregnated with *Metarhizium anisopliae* conidia, with and without Atr*Aedes* lures

Treatments/Time	Black cloth + fungus + lure	Black cloth + fungus	Black cloth + lure	Black cloth only
120 h	33.3 ± 0.6 A a	47.3 ± 0.6 B a	82.6 ± 1.5 C a	79.3 ± 0.6 C a
72 h	34.0 ± 2.6 A a	56.0 ± 3.5 B a	81.3 ± 2.1 C a	78.6 ± 2.1 C a
48 h	36.6 ± 1.5 A a	65.3 ± 1.5 B b	80.6 ± 1.5 C a	82.0 ± 1.0 C a
24 h	38.6 ± 0.6 A a	76.6 ± 2.9 B c	78.6 ± 0.6 B a	81.3 ± 1.2 B a

Results followed by different capital letters indicate significant differences between mosquito survival rates when comparing different treatments (lines). Results followed by different small letters indicate significant differences between mosquito survival rates when comparing different exposure times using the same treatment (columns). Statistical differences were analysed using a one-way ANOVA and Duncan’s *post-hoc* test at the 5% level

## Discussion

Although the use of entomopathogenic fungi for controlling adult mosquitoes appears promising under laboratory and simulated field conditions, much research is still needed to effectively deliver these agents to their targets or to attract the targets to the control agent.

We have previously shown that exposing mosquitoes to fungus-impregnated black cloths hung in large cages under simulated field conditions effectively reduced adult *Aedes* survival [9, 18]. Furthermore, our group has also shown that black cloths are highly attractive to female *Ae. aegypti* and that impregnating these cloths with conidia of the entomopathogenic fungus *M. anisopliae* does not reduce their attractiveness [19]. We have also proven the effectiveness of these fungus-impregnated cloths at reducing mosquito survival in rooms simulating human residences when attached to items of furniture using silver tape [19].

We recently developed a new fungus deployment strategy which does not involve fixing the black cloths to furniture using adhesive tape. Fungus-impregnated cloths were suspended inside two liter plastic bottles which had one side cut away to allow the mosquitoes easy access to land on the cloths [17]. This so called “PET trap” could be quickly and simply deployed and subsequently collected from residences when it was time to renew the traps. The PET traps were equally effective at reducing mosquito survival when impregnated with conidia of either *M. anisopliae* or *B. bassiana* [17]. When deployed in observation chambers, it was necessary to leave the traps for at least 24 h in the presence of the mosquitoes to cause significant reduction in mosquito survival. Lower exposure times did not significantly reduce mosquito survival when compared to controls. Both fungal species were effective at reducing survival rates when mosquitoes were exposed to traps for either 24 h or 48 h under intra-domicile conditions and the results showed that five traps or three traps per room were equally effective in reducing mosquito survival rates; however, one PET trap per room was not as efficient [17].

A recent study showed that very short exposure times (1 minute) of *Anopheles stephensi* adults to cloths impregnated with *B. bassiana* conidia were sufficient to cause high levels of infection [20]. The results seen by Silva et al. [17] showed no significant reduction in survival rates of mosquitoes exposed to traps impregnated with *M. anisopliae* or *B*. *bassiana* over a 12 h period in observation chambers. This would appear to indicate a lower virulence of the isolates used in our study or a higher susceptibility of *An. stephensi* to fungal infection than *Ae. aegypti*. However, the experiments carried out by George et al. [20] were performed in olfactometers to demonstrate the attraction of the insects to the fungus-impregnated cloths and that subsequently a one minute contact with these cloths resulted in a 95% infection rate. In our experiments, the mosquitoes may not have landed on the cloths during this 12 h period.

Fungi did not repel *Ae. aegypti* [19] and interestingly were even shown to be attractive to *An. stephensi* [20]. Our work showed no differences in landing frequencies on fungus- or non-fungus-impregnated black cloths. Although insects such as mosquitoes could be naturally attracted to entomopathogenic fungi, one strategy to increase infection rates and reduce the need for deploying large numbers of fungus-impregnated landing surfaces, is the use of attractive lures. Here we tested the combination of fungus-impregnated cloths with a commercially available attractive lure, Atr*Aedes*.

However, before testing a combination of fungus-impregnated black cloths in PET traps + Atr*Aedes*, we first established the attractiveness of the lures under intra-domicile conditions (Table 1). Black cloths were covered in adhesive film to monitor the landing/capture frequency over different time periods (24 and 48 h). PET traps with adhesive film only captured very low numbers of mosquitoes, showing that occasionally, mosquitoes can land on the sticky films, but they are not attracted to the PET traps *per se*. Increased capture rates were observed when using Atr*Aedes* together with adhesive films. In this case, 10% of the mosquitoes released into the rooms were captured during the first 24 h and this increased to 22% at 48 h. PET traps with black cloths covered in adhesive films were actually more attractive to mosquitoes than adhesive films + Atr*Aedes*, although the capture rates were not statistically different at 24 h (Table 1). This experiment shows the importance of using a dark surface to attract *Ae. aegypti*. PET traps were then tested with a combination of black cloths covered in adhesive films and with an Atr*Aedes* lure attached to the base of the cloth. This combination resulted in the highest capture rates recorded here, with 31% of mosquitoes captured at 24 h and 66% at 48 h. These results were significantly different to all the other treatments. The results affirm the importance of using two types of attractive cues, visual and olfactory. Black surfaces are known to attract a range of mosquito species, including *Ae. aegypti* [21].

The use of odor-baited stations containing fungus-impregnated cloths was tested against the malaria mosquito *Anopheles arabiensis*, when positioned in rural areas away from human dwellings [22]. The results showed that 95% of mosquitoes that visited the fungus- impregnated bait stations died within 14 days. In this case, a yeast-based lure was used to generate CO_2_ which attracted the mosquitoes.

The Atr*Aedes* lure was initially developed for use in the MI-Dengue system (ECO-VEC Ltd., Belo Horizonte, Brazil), deployed in adult traps called MosquiTRAP™ to monitor mosquito populations. This synthetic lure was developed from research investigating attractive substances in volatile grass infusions [23]. These lures have been stated to effectively attract *Ae. aegypti* for at least 45 days [23], although these results have not been published. Our results show a slow decline in attractiveness of the lures over time, with the maximum time tested being 30 days. However, lures continued to attract *Ae. aegypti* at this time.

Although the Atr*Aedes* lure is easy to use and much more convenient than grass infusions, it did not attract gravid *Culex quinquefasciatus* in search of oviposition sites when tested in Tanzania [24], even though this species has been found in Atr*Aedes*-baited MosquiTRAPs in Brazil [25]. In that case, *Cx. quinquefasciatus* could have been attracted to enter the MosquiTRAP independent of the lure, as these traps are attractive to mosquitoes *per se*.

In a study comparing the efficiency of three different traps for the capture of three mosquito species, *Cx. quinquefasciatus*, *Ae. aegypti* and *Aedes simpsoni* in northeastern Tanzania, the CDC-Gravid Trap caught significantly more *Cx. quinquefasciatus* and *Ae. aegypti* than the sticky ovitrap or MosquiTRAP, but there was no significant difference in the number of mosquitoes caught between the two sticky traps or of *Ae. simpsoni* (*s.l.*) caught when comparing the three trap types [26]. In fact, the lower capture rates of the two sticky traps are compensated by their low cost when compared to the expensive and complicated to use CDC-Gravid Trap. An even lower cost trap has now been developed to monitor mosquito populations. The passive Gravid *Aedes* Trap (GAT) which is commercial available from Biogents Ltd., captures gravid *Aedes* without the use of adhesives commonly used in lethal ovitraps or electrically powered fans and lights such as used in the CDC-Gravid Trap and the BG-Sentinal [27]. The GAT is a useful tool for capturing adult *Ae. aegypti* and may be suitable for other container-inhabiting species such as *Aedes albopictus* and *Cx. quinquefasciatus*. The GAT should be used in conjunction with insecticides or alternative compounds which impede the mosquitoes from escaping [28].

Taking low cost and ease of use into consideration, we developed our PET Trap system. Although we have already demonstrated proof of concept, there is always room for improvement. In the current study we have shown the increased efficiency of fungus-impregnated PET traps when combined with the Atr*Aedes* lure under simulated intra-domicile conditions. It was possible to significantly reduce the survival rates of *Ae. aegypti* females when using only one PET trap per test room. Our previous study deployed one, three or five PET traps per room [17]. There was no difference between three and five traps but a significant increase in survival was observed when using one trap per room. However, the reduction in survival when using one PET trap per room (approx. 45%) was still significantly lower than the controls (80%). Here we obtained improved results when using only one PET trap per room in combination with the Atr*Aedes* lure (Table 2, 32% mean survival). The significant reduction in survival when combining PET traps with Atr*Aedes* is an important finding. Although we did not observe the number of mosquitoes landing on the fungus-impregnated cloths in the presence or absence of the lure, the results for capture of mosquitoes when using adhesive films leads us to believe that higher numbers of mosquitoes become infected by the fungus when using the lures due to higher landing rates on cloths and possibly longer time periods resting on the cloths.

We are currently testing PET traps impregnated with entomopathogenic fungi in human residences. These tests involve the distribution of 15 traps with fungi and 15 control traps, which are randomly distributed to covered verandas of ground floor apartments. The possible effects on the mosquito population are being monitored using ovitraps to estimate the number of eggs being laid by *Ae. aegypti* on a weekly basis, in the presence or absence of the fungi. The results are promising, with a significant reduction in egg counts in ovitraps placed in the vicinity of the fungus-impregnated PET traps when compared to controls (unpublished data).

We have yet to test the PET traps with Atr*Aedes* in human residences but believe that this will result in further decreases in egg counts due to increased infection of *Ae. aegypti* females. We have shown the effectiveness of the lures in attracting mosquitos over long time periods and believe it will be possible to leave the PET traps for up to one month in human dwellings.

## Conclusions

We confirmed increased attractiveness of PET traps with black cloths under intra-domicile conditions when associated with Atr*Aedes*. The use of attractive synthetic lures associated with black cloths impregnated with *M. anisopliae* or *B. bassiana* conidia significantly reduced *Ae. aegypti* survival in simulated intra-domicile conditions when compared to traps with fungus-impregnated cloths only. The attractiveness of the lures was only slightly reduced over time. The use of fungus-impregnated cloths is a promising application method for the control of *Ae. aegypti* and the use of attractive lures further increases efficiency of this method, significantly reducing the survival of the mosquitoes and reducing the number of PET traps necessary per household.

## Additional files

Additional file 1: PET Traps with black cloths, adhesive films and attractive lures. **Figure S1** PET traps. **a** PET trap with black cloth only. **b** PET trap with adhesive film and attractive lure. **c** PET trap with black cloth covered in adhesive film and attractive lure attached to the base of the cloth. (PDF 424 kb)

Additional file 2: Test rooms used in experiments. **Figure S2** Details of the test rooms used in experiments simulating intra-domicile conditions. **a** External view of the four identical test rooms. **b** Internal arrangement of test room. **c** Ceiling extractor fan used to circulate air within the room. (PDF 628 kb)

## Abbreviations

RH: Relative humidity
TW: Tween 80
