# Supplementary figures and images for: Improving the delivery and efficiency of fungus-impregnated cloths for control of adult Aedes aegypti using a synthetic attractive lure
Source: Parasit Vectors. 2018 May 4;11:285. doi: 10.1186/s13071-018-2871-z (PMC5936027; doi:10.1186/s13071-018-2871-z)

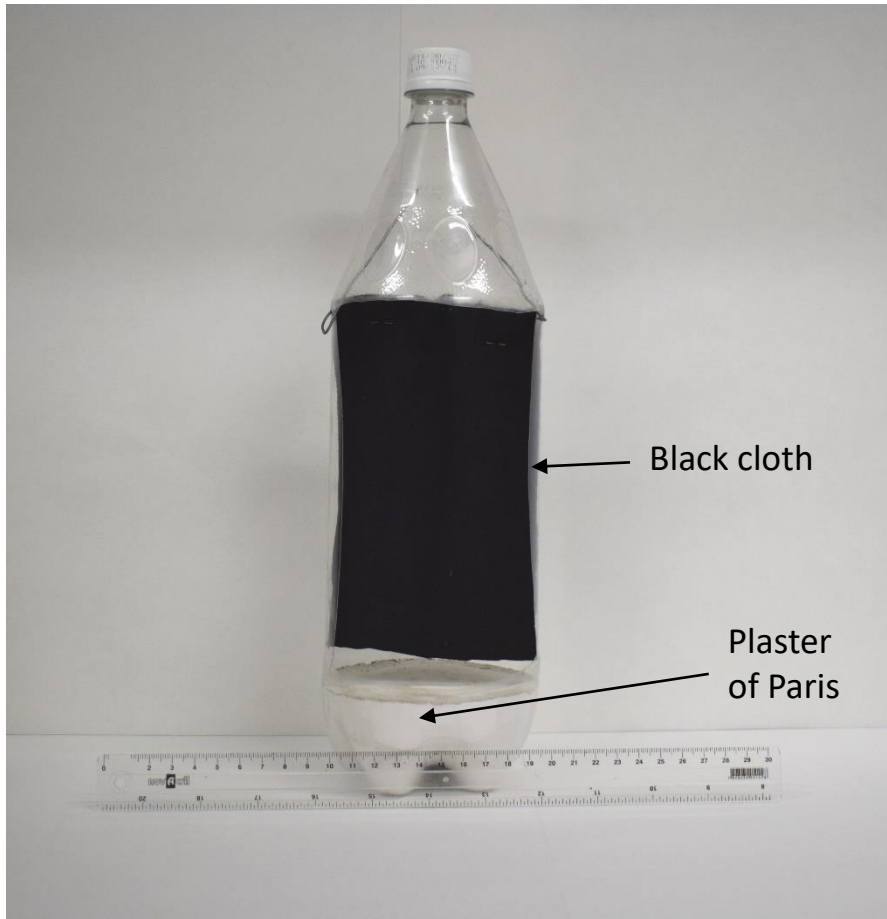

Figure S1 a

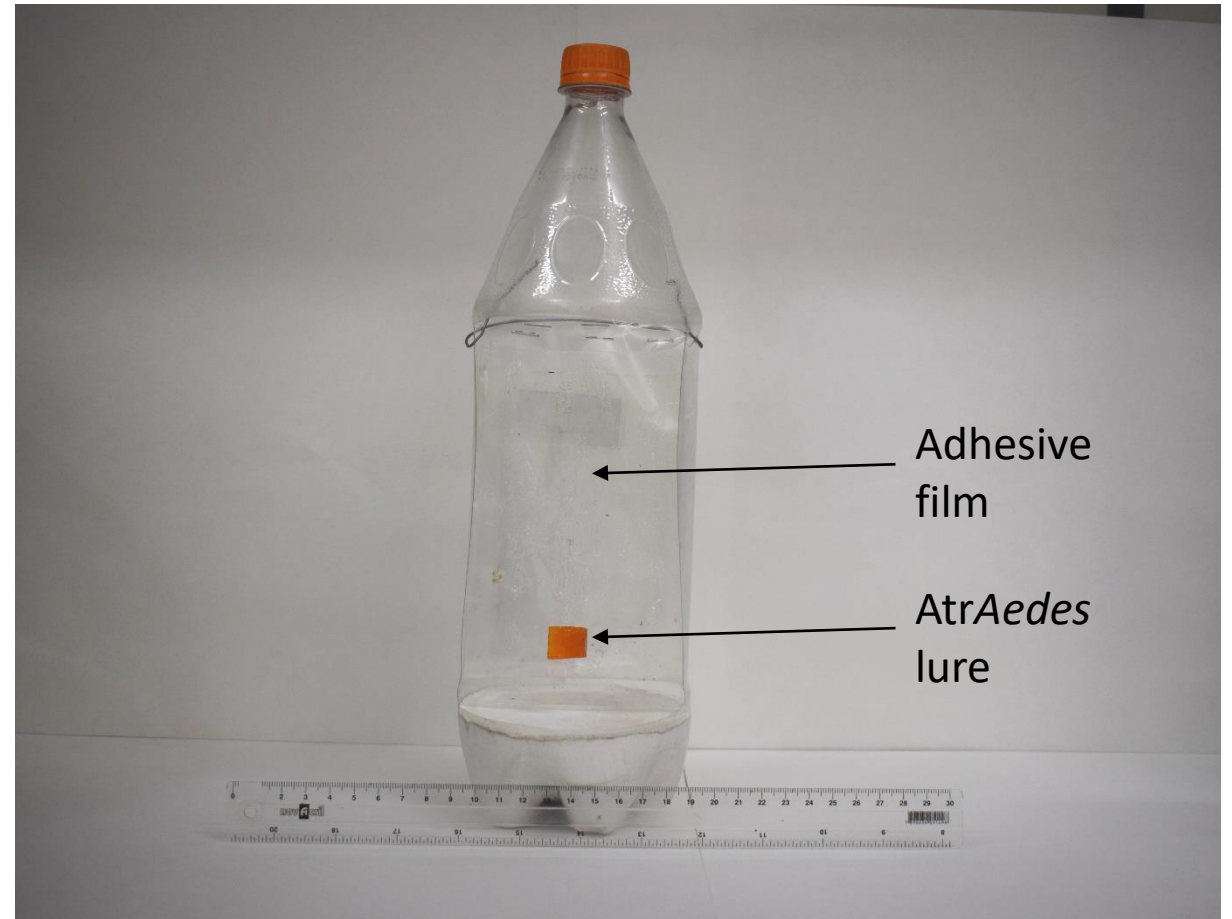

Figure S1 b

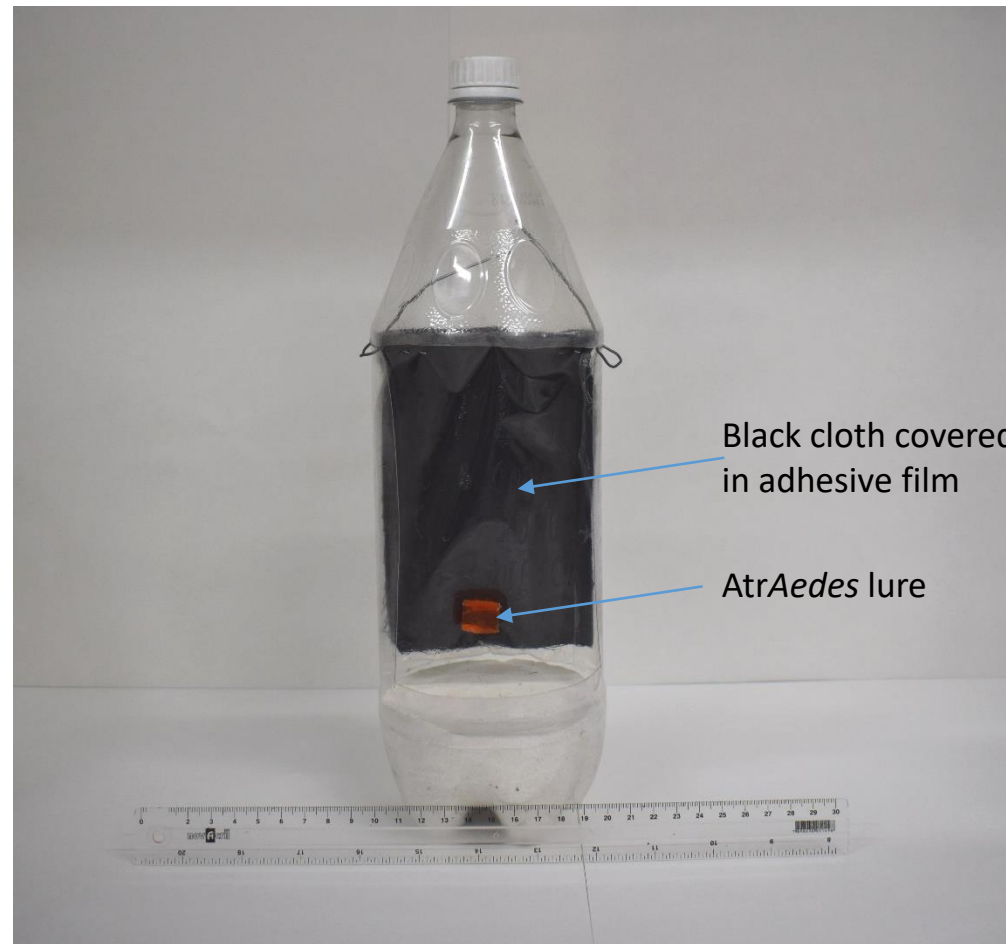

Figure S1 c

Supplement: Supplementary file 1 — PET Traps with black cloths, adhesive films and attractive lures. Figure S1 PET traps. a PET trap with black cloth only. b PET trap with adhesive film and attractive lure. c PET trap with black cloth covered in adhesive film and attractive lure attached to the base of the cloth. (PDF 424 kb) [file 13071_2018_2871_MOESM1_ESM.pdf]
