# Supplementary material for: Improving the delivery and efficiency of fungus-impregnated cloths for control of adult Aedes aegypti using a synthetic attractive lure
Source: Parasit Vectors. 2018 May 4;11:285. doi: 10.1186/s13071-018-2871-z (PMC5936027; doi:10.1186/s13071-018-2871-z)
Supplement: Supplementary file 2 — Test rooms used in experiments. Figure S2 Details of the test rooms used in experiments simulating intra-domicile conditions. a External view of the four identical test rooms. b Internal arrangement of test room. c Ceiling extractor fan used to circulate air within the room. (PDF 628 kb) [file 13071_2018_2871_MOESM2_ESM.pdf]

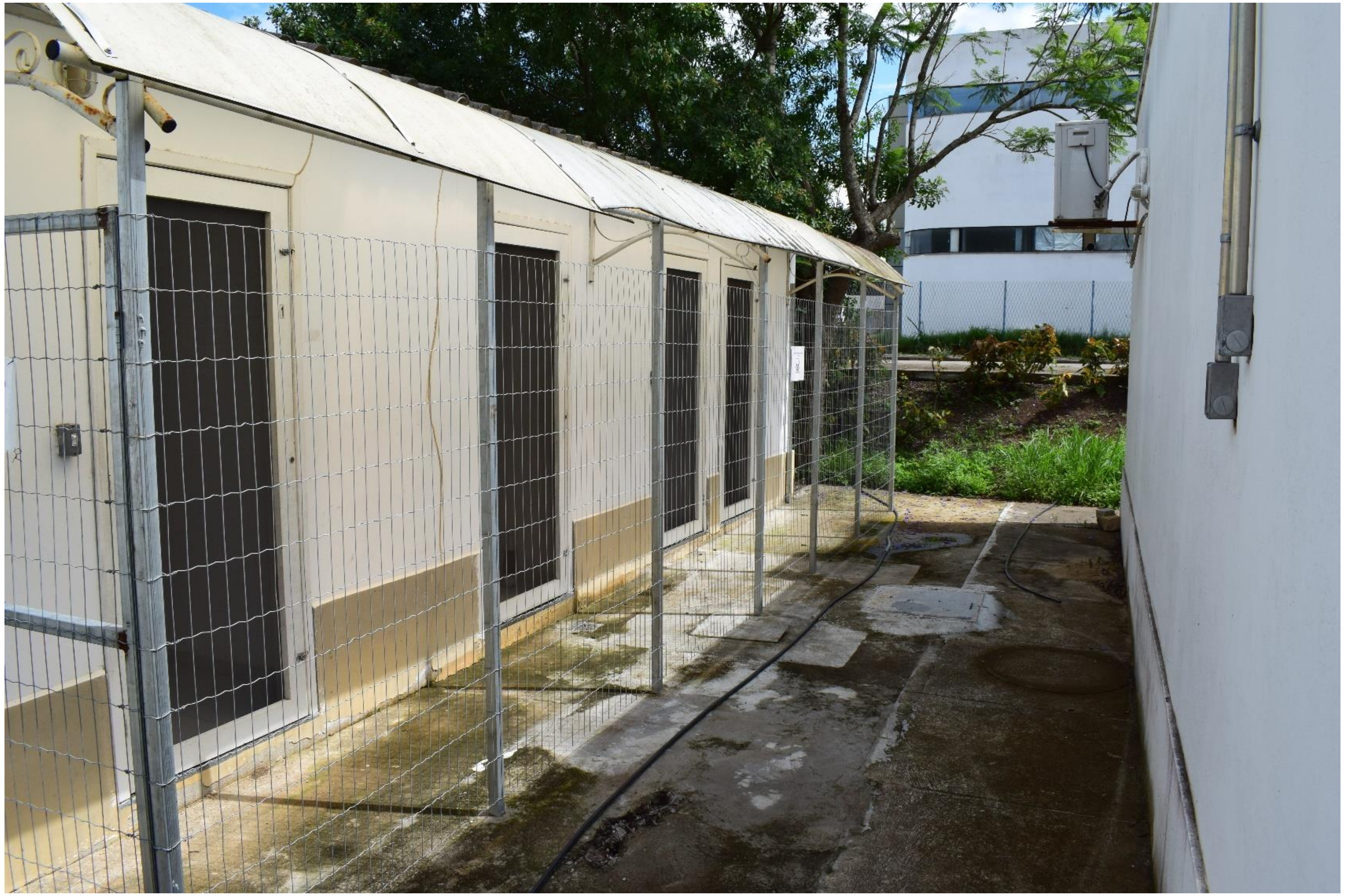

Figure S2a: Four identical test used in experiments

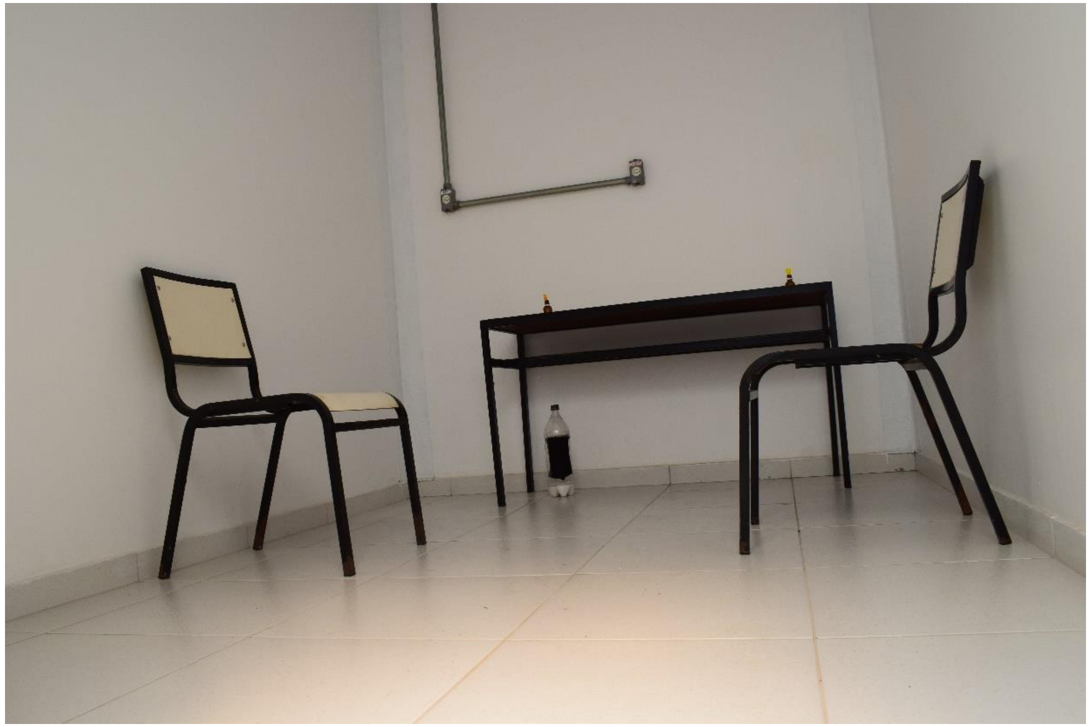

Figure S2b: Internal arrangement of test room with PET trap placed on the floor and wick feeders on the table.

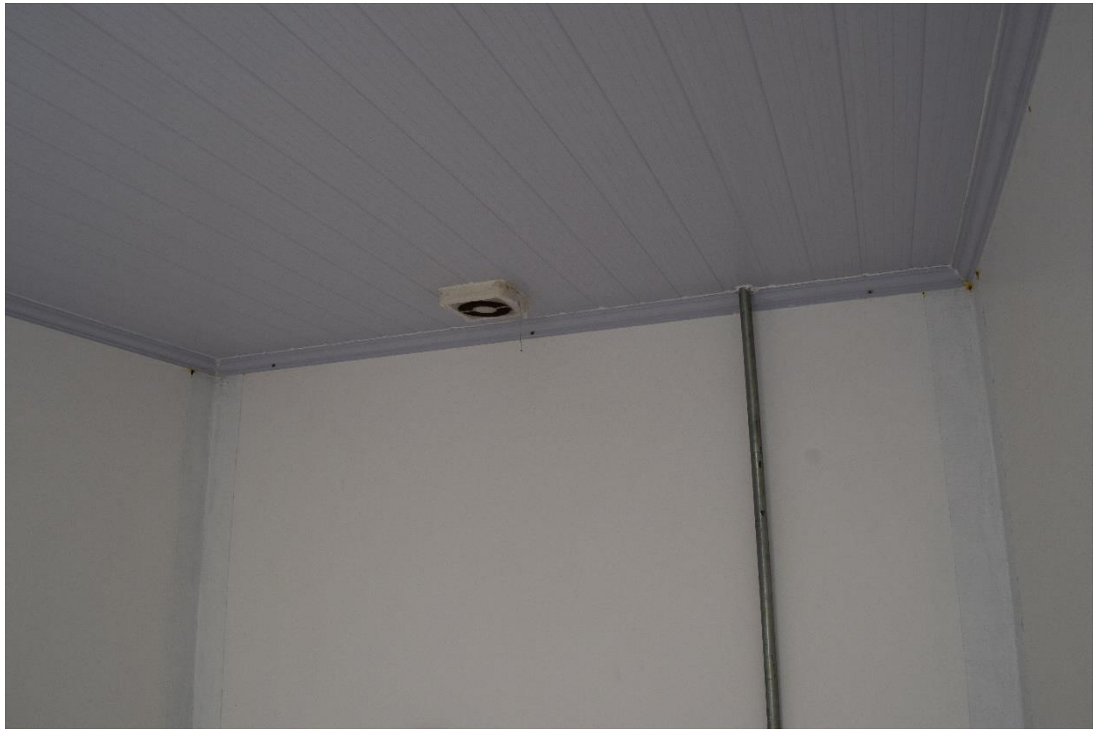

Figure S2c: Extractor fan in the ceiling to create a constant air flow in the test room. The entrance to the fan was covering in netting to prevent mosquitoes escaping.
